# LRP5, Bone Density, and Mechanical Stress: A Case Report and Literature Review

**DOI:** 10.3389/fendo.2019.00184

**Published:** 2019-03-26

**Authors:** Nicholas G. Norwitz, Adrian Soto Mota, Madhusmita Misra, Kathryn E. Ackerman

**Affiliations:** ^1^Department of Physiology, Anatomy and Genetics, University of Oxford, Oxford, United Kingdom; ^2^Harvard Medical School, Boston, MA, United States; ^3^Division of Pediatric Endocrinology, Massachusetts General Hospital, Boston, MA, United States; ^4^Neuroendocrine Unit, Massachusetts General Hospital, Boston, MA, United States; ^5^Divisions of Sports Medicine and Endocrinology, Boston Children's Hospital, Boston, MA, United States

**Keywords:** bone mineral density, LRP5, mechanical stress, osteoporosis, Wnt-β-catenin signaling

## Abstract

The Wnt-β-catenin pathway receptor, low-density lipoprotein receptor-related protein 5 (LRP5), is a known regulator of bone mineral density. It has been hypothesized that specific human polymorphisms in *LRP5* impact bone density, in part, by altering the anabolic response of bone to mechanical loading. Although experiments in animal models support this hypothesis, there is limited evidence that *LRP5* polymorphisms can alter the anabolic response of bone to mechanical loading in humans. Herein, we report a young male who harbors a rare *LRP5* missense mutation (A745V) and who provides potential proof of principle for this mechanotransduction hypothesis for low bone density. The subject had no history of fractures until age 18, a year into a career in competitive distance running. As he continued to run over the following 2 years, his mileage threshold to fracture steadily and rapidly decreased until he was diagnosed with severe osteoporosis (lumbar spine BMD Z-score of −3.2). By contextualizing this case within the existing *LRP5* and mechanical stress literature, we speculate that this represents the first documented case of an individual in whom a genetic mutation altered the anabolic response of bone to mechanical stress in a manner sufficient to contribute to osteoporosis.

## Background

**Figure 1:**
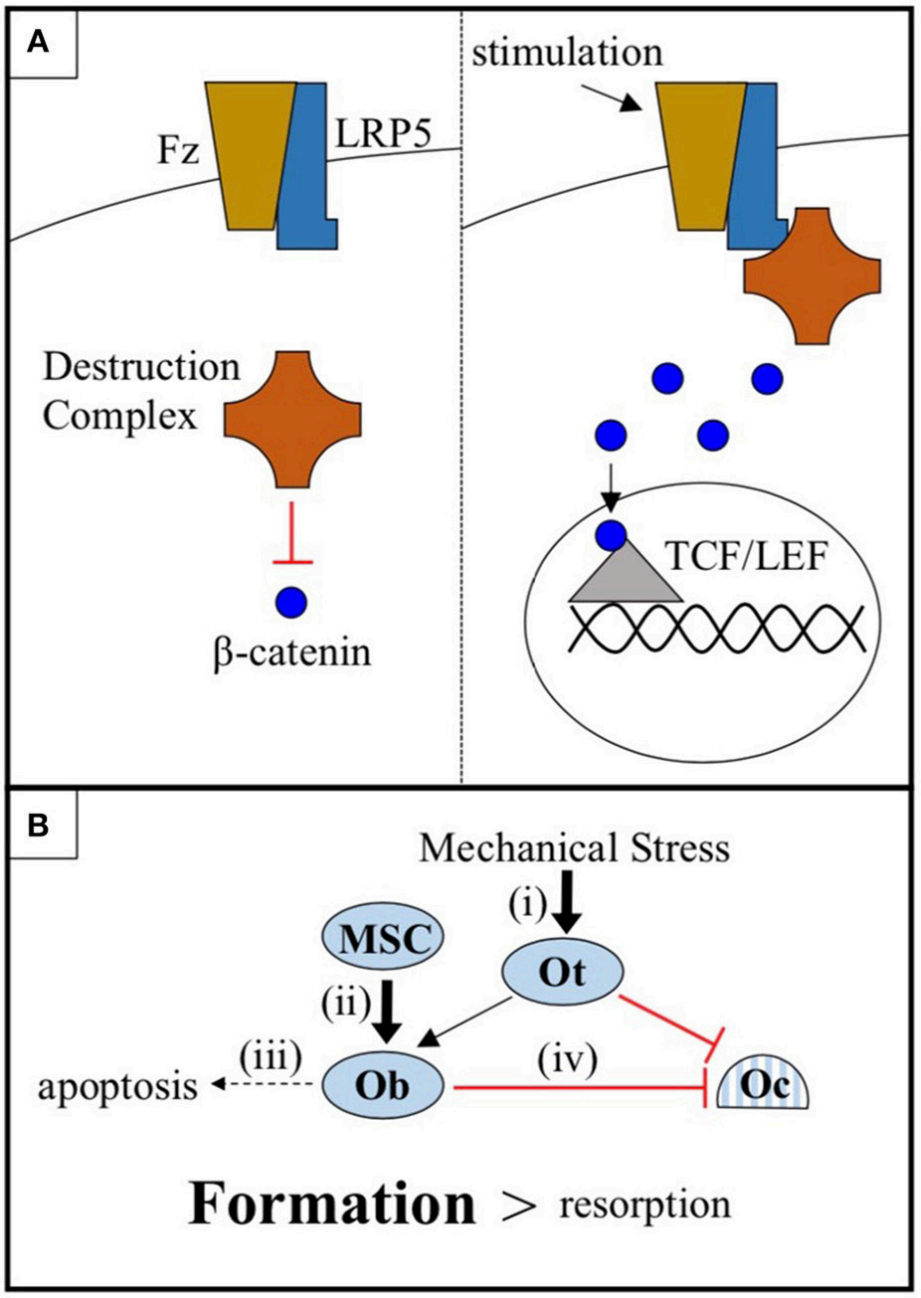
**(A)** The cytoplasmic destruction complex constitutively targets β-catenin for degradation. Mechanical or chemical stimulation of the Wnt-β-catenin pathway receptor pair LRP5-Frizzled (Fz), in cells of the osteoblast linage, causes LRP5 to sequester the destruction complex, allowing β-catenin to accumulate and translocate to the nucleus where it interacts with TCF/LEF family transcription factors and promotes osteogenic gene expression. **(B)** Osteocytes (Ot) sense mechanical stress and respond by increasing Wnt-β-catenin signaling and coordinating the anabolic activities of osteoblasts (Ob) and the catabolic activities of osteoclasts (Oc). Wnt-β-catenin signaling, in addition to (i) sensitizing osteocytes to mechanical stress, (ii) promotes the differentiation of mesenchymal stem cells (MSC) into osteoblasts, (iii) prevents osteoblast apoptosis, and (iv) increases osteoprotegerin expression by osteoblasts, thus inhibiting osteoclast-mediated bone resorption. The net effect is a shift in favor of bone formation over bone resorption.

Low-density lipoprotein receptor-related protein 5 (LRP5) is a 1,615 amino acid transmembrane receptor for the conserved Wnt-β-catenin signaling pathway, a pathway known to regulate bone metabolism in humans. In canonical Wnt-β-catenin signaling, a Wnt ligand binds to a binding site created by the 1^st^ and 3^rd^ β-propeller domains of LRP5 and to its co-receptor, Frizzled. This enables LRP5 to sequester a cytoplasmic destruction complex and, thereby, prevent the degradation of the protein β-catenin. Subsequently, β-catenin translocates into the nucleus, where it interacts with TCF/LEF family transcription factors and alters gene expression to promote bone formation (1) (Figure 1A). Genome-wide association studies (GWAS) have repeatedly classified *LRP5* as a key mediator of bone mineral density (BMD) (2–4), including the largest GWAS to date, which identified *LRP5* as a BMD and fracture risk locus at a significance level of p < 1.0 x 10^−21^ (5).

*LRP5* mutations are known to cause disorders of both low and high BMD. Recessive loss-of-function mutations in *LRP5* cause osteoporosis-pseudoglioma syndrome (OPPG), a condition characterized by severe osteoporosis and occasional ocular abnormalities (1, 6), whereas gain-of-function mutations in *LRP5* are associated with abnormally high BMD (7). Furthermore, *LRP5* demonstrates haploinsufficiency (6, 8–11). In fact, dominant loss-of-function mutations in *LRP5* are among the most common causes of familial exudative vitreoretinopathy (FEVR), a congenital eye defect that often presents with a comorbid low BMD phenotype (10, 11). Of note, *LRP5* haploinsufficiency appears to affect BMD in men more severely than in women (12–15). In addition to GWAS and clinical associations, *LPR5* heterozygous (*LPR5*^+/−^) mouse models reliably exhibit low BMD (16–18). Consistent with data from human studies, the loss-of-function phenotype is more severe in male mice than in female mice, with male mice exhibiting lower relative BMDs, shortened femurs during their youth, and a reduced osteogenic response to mechanical stress (17).

There are a number of mechanisms by which LRP5-mediated Wnt-β-catenin signaling in cells of the osteoblast lineage may promote bone growth. These include (i) sensitizing osteocytes to mechanical stress, (ii) promoting the differentiation of mesenchymal stem cells (MSC) into osteoblasts, (iii) preventing osteoblast apoptosis, and (iv) increasing osteoblast expression of osteoprotegerin to decrease osteoclastogenesis (19–24) (Figure 1B). While each of these mechanisms likely plays a part in mediating the regulatory effects of the LRP5 protein on BMD, the mechanical stress model (i) is the focus of this report. There is an abundance of mouse data to support this model. First, *LRP5* gain-of-function mutations in mice do not appear to increase basal rates of bone formation in the absence of mechanical stimulation, but more than double bone formation in response to mechanical stress (25, 26). Second, *LRP5* gain-of-function enhances the expression of bone formation genes in response to mechanical stress (24). Third, conditional knockout of *LRP5* in murine osteocytes, cells which are believed to serve as the mechanosensors of bone, diminishes the osteogenic response to mechanical stress, whereas activation of Wnt-β-catenin signaling in osteocytes is sufficient to increase the osteogenic response (27–29). Thus, data from mice support a model in which the LRP5 receptor influences BMD, at least in part, by regulating mechanotransduction.

Three clinical observations of patients bearing *LRP5* mutations also support the mechanical stress model. First, *LRP5* mutations do not appear to affect calcium homeostasis, anabolic or catabolic hormones, collagen synthesis, or basal levels of bone turnover, even in patients with severe osteoporosis (9, 13). Second, *LRP5* gain-of-function mutations can increase BMD without affecting bone shape or causing bony lesions, which are observed in genetic conditions that simply increase basal osteoblast activity or decrease basal osteoclast activity (30). Third, *LRP5* gain-of-function mutations cause the greatest enhancement of BMD in load bearing bones (30).

Two population-based studies add yet another level of support to the mechanical stress model. In a subset of 868 men from the Framingham Offspring Study Cohort, a polymorphism in exon 10 of *LRP5* appeared to negatively affect the interaction between physical activity and BMD. Specifically, men homozygous for the common allele exhibited a positive correlation between physical activity and BMD; heterozygous men exhibited no correlation; and men homozygous for the less common allele exhibited a negative correlation between physical activity and BMD (31). Similar data were reported from the Odense Androgen Study. In this study of 783 men aged 20–30, the *LRP5* polymorphisms A1330V and V667M were associated with low BMD in physically active men, but not in sedentary men (32). Although these two independent studies each suggest that polymorphisms in *LRP5* can alter the anabolic response of bone to mechanical stress in men, they were limited by the fact that they assessed physical activity using questionnaires.

We report a 23-year-old male ex-distance runner who presented with primary osteoporosis and a rare *LRP5* variant, A745V in exon 10, at age 20. His mutation, medical history, and athletic history complement and build upon the mouse models, clinical observations, and epidemiological data introduced above. In brief, this case represents potential proof of principle for the mechanical stress model and suggests the possibility that *LRP5* mutations contribute to low BMD, in part, by blunting the anabolic response of bone to mechanical stress.

## Case Report

**Figure 2:**
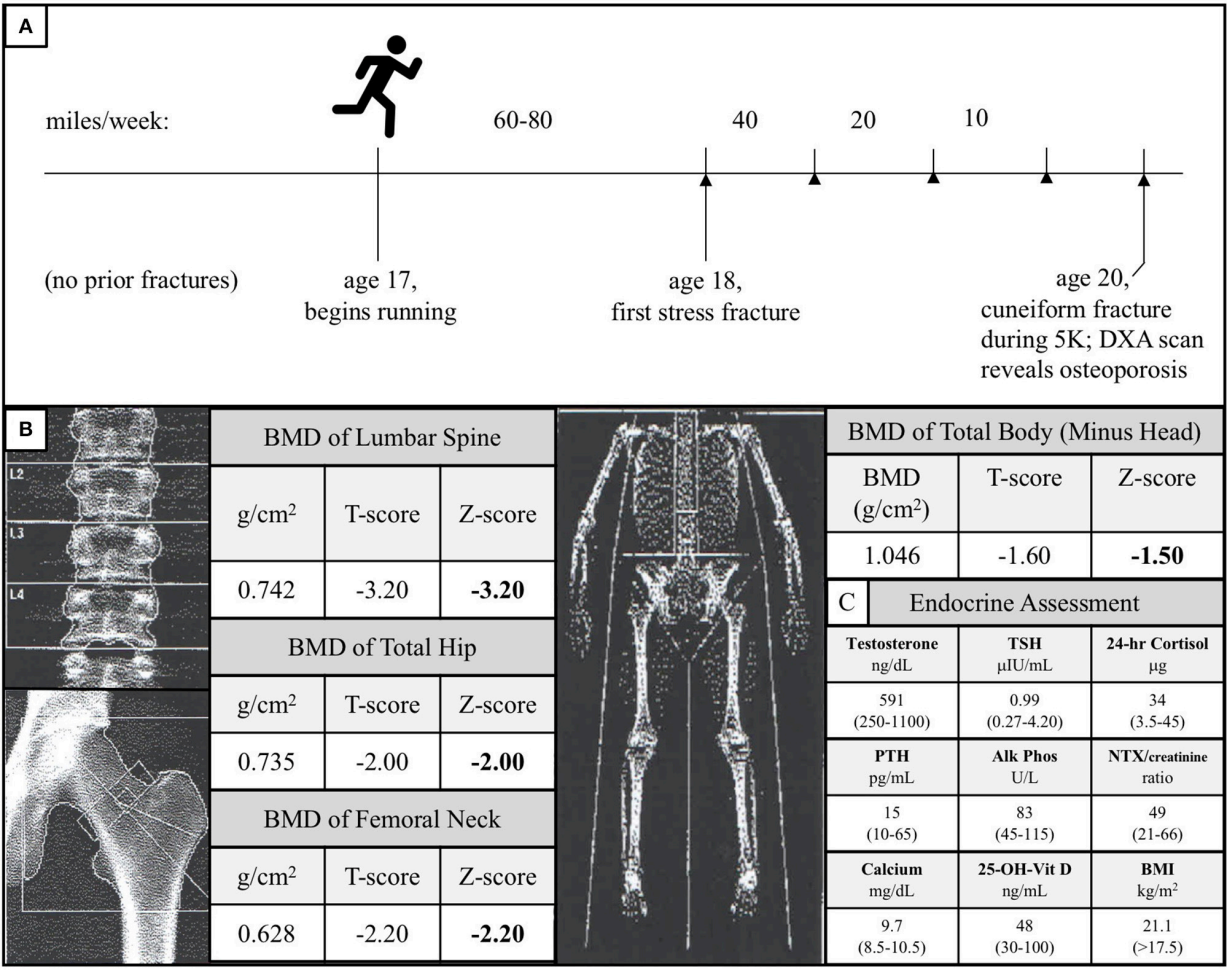
**(A)** Diagram of the subject's running and stress fracture history. The subject had no history of fractures during his childhood and began distance running at age 17. He successfully ran 60–80 miles per week for over 1 year before experiencing his first fracture in his right lateral tibial plateau. Over the subsequent years, as he continued to run, his mileage threshold to fracture decreased precipitously (stress fractures are represented by arrowheads). At age 20, he fractured his right cuneiform during a 5-kilometer road race. A follow-up of the unusual foot fracture revealed osteoporosis. **(B)** DXA scan of the subject's lumbar spine, total hip, femoral neck, and total body (minus head) at time of diagnosis. These data are consistent with the notion that the subject's load-bearing bones failed to adapt to the mechanical stress of running. **(C)** Endocrine assessment at time of diagnosis. Reference ranges are given in parentheses and BMI >17.5 kg/m^2^ is used because this threshold is a surrogate marker for Relative Energy Deficiency in Sport (RED-S) in men (33, 34).

The Caucasian male subject was the product of an uncomplicated pregnancy, although he did exhibit shortened femurs *in utero*, similar to *LRP5* loss-of-function male mice (17). He demonstrated no signs of any chronic health condition during his highly active youth or adolescence, during which he engaged in a variety of sports, including basketball, soccer, rugby, and martial arts. He began competitive distance running at age 17. For over 1 year, he consistently ran between 60 and 80 miles per week without sustaining any bone injuries. At age 18, he sustained a stress fracture in his right lateral tibial plateau. Subsequent to this initial stress fracture, he began to experience stress fractures at progressively lower mileage thresholds. After fracture resolution, physical therapy, and a gradual return to running, he sustained further tibial, femoral, and sacral alar stress fractures when running 40, 20, and even 10 miles per week, consistent with the notion that his bones were weakening as he continued to run (Figure 2A).

At age 20, he sustained a complete fracture of his right cuneiform during a 5-kilometer run. A dual-energy X-ray absorptiometry (DXA) scan was performed given this history of recurrent fractures and this revealed a lumbar spine BMD Z-score of −3.2, total hip BMD Z-score of −2.0, femoral neck BMD Z-score of −2.2, and total body (minus head) BMD Z-score of −1.5 (Figure 2B). At the time of diagnosis, the subject had a normal BMI (21.1 kg/m^2^), normal resting metabolic rate (1,613 kcal/day, measured by respirometry vs. 1,604 kcal/day, calculated using the Harris-Benedict equation), normal testosterone, TSH, 24-h urine free cortisol, PTH, alkaline phosphatase, urinary N-terminal telopeptide/creatinine, calcium, and 25-OH-Vitamin D (Figure 2C). All other electrolytes, hormones, and kidney and liver function tests were unremarkable, and the subject, now 23, has exhibited no meaningful signs of endocrine dysfunction in the years since initial evaluation.

A genetic screen revealed an undocumented paternally-inherited polymorphism (A745V) in the *LRP5* gene. His father, a 54-year-old with a BMI of 37.2 kg/m^2^, did not exhibit low BMD at the lumbar spine, total hip, or femoral neck (T-scores of 0.0, 0.9, and 0.1, respectively); however, the father did exhibit a radial BMD T-score of −2.6 (age-adjusted Z-score of −2.0). The subject completed a 13-month course of teriparatide, which increased his lumbar spine BMD Z-score from −3.2 to −2.7, and he is currently on denosumab.

The subject's only other health condition is ulcerative colitis, which was diagnosed at age 22, 4 years after his first fracture. The colitis is mild and localized to the cecum and sigmoid colon. As the subject never exhibited evidence of malabsorption/malnutrition or systemic inflammation (his high sensitivity CRP was consistently measured to be low both before and after his colitis diagnosis), was never on chronic glucocorticoids, and had absolutely no symptoms of this condition at the time that he was having the fractures, it is unlikely that his ulcerative colitis contributed to his low BMD. This opinion was unanimously shared by three independent gastroenterological consults.

## Discussion

**Figure 3:**
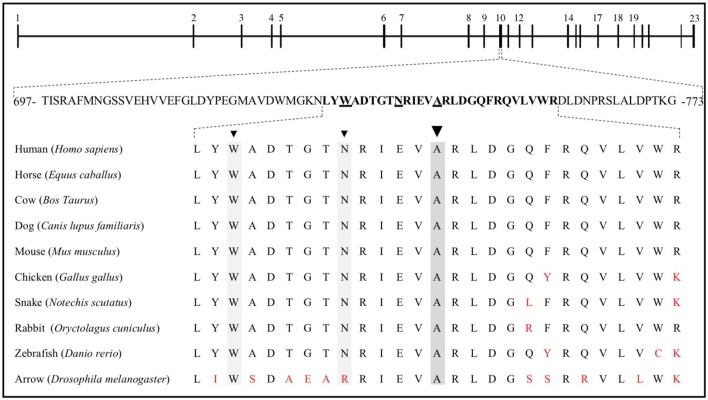
The *LRP5* gene is composed of 23 exons, coding for 1,615 amino acids. Exon 10 includes residues 697 to 773, 27 of which are sequence aligned with the corresponding horse, cow, dog, mouse, chicken, snake, rabbit, and zebrafish *LRP5* sequences, as well as with that of the homologous protein in Drosophila, arrow. W734 (6), N740 (31), and A745 are underlined and identified by arrowheads. Red letters represent nonconserved residues.

**Figure 4:**
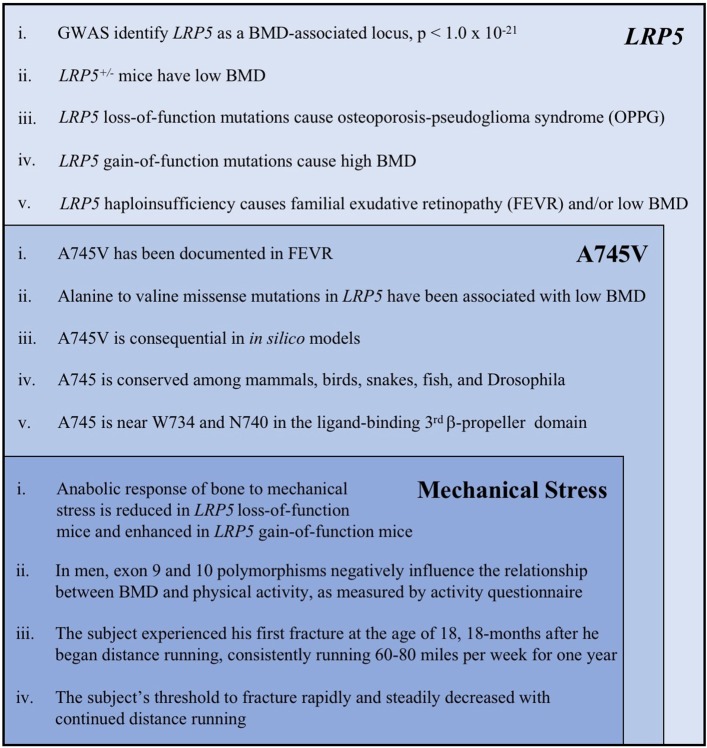
A summary of the key evidence supporting the role of *LRP5*, the A745V variant, and mechanical stress in the etiology of the subject's osteoporosis. Each category of evidence builds upon, and is inset within, the previous category. The following references correspond to each line of evidence: ***LRP5***(i). Trajanoska et al. (5) (ii). Sawakami et al. (17), Clement-Lacroix et al. (16), Yadav et al. (18) (iii). Gong et al. (6), Joiner et al. (1) (iv). Johnson et al. (30), Johnson (7) (v). Toomes et al. (11), Qin et al. (10). **A745V** (i). Pefkianaki et al. (35) (ii). Brixen et al. (32), Estrada et al. (3) (iii). Pefkianaki et al. (35) (iv). NCBI sequence analyzer and alignment tools were used to assess conservation (v). Gong et al. (6), Joiner et al. (1). **Mechanical Stress** (i). Sawakami et al. (17), Zhao et al. (29), Robinson et al. (24), Johnson (7), Niziolek et al. (25) and others (see text) (ii). Brixen et al. (32), Kiel et al. (31) (iii, iv). Information from the subject's medical history.

The properties of the A745V variant suggest that it likely contributed to the subject's osteoporosis. A745V is extremely rare, with a minor allele frequency of 0.0008 in the Genome Aggregation Database (254/282476 alleles; 0 homozygotes), and is perfectly conserved among mammals, birds, snakes, fish, and even the Drosophila homolog of *LRP5*. It is located within the Wnt-ligand-binding 3^rd^ β-propeller domain, adjacent to two other residues (N740, from the Framingham Study, and W734) mutations in which are also associated with low BMD in humans (6, 31) (Figure 3). Other alanine to valine missense mutations in *LRP5* have been reported to contribute to low BMD. The A745V variant was predicted to be consequential in *in silico* models and was reported to contribute to a case of FEVR, which is often associated with low BMD (3, 32, 35) (Figure 4).

Despite the evidence supporting the consequence of the A745V variant mentioned in the previous paragraph, the proposition that this inherited genetic mutation was a major contributor to the subject's osteoporosis raises two important questions: (1) Why is there a discrepancy between the subject's BMD and that of his father? (2) If the subject's low BMD is attributable to a congenital genetic defect, why did it only manifest with fractures over 1 year into his running career when he was a young adult? The discrepancy between the father's and son's BMDs may be explained, in part, by the variable expressivity observed repeatedly with *LRP5* mutations. In the first report of this A745V variant, the carrier father exhibited only subclinical symptoms (35); and probands heterozygous for inherited *LRP5* mutations often exhibit BMDs significantly lower than those of their carrier parents (8, 9). It is also possible that the father's higher BMI (37.2 kg/m^2^) was somewhat protective for his BMD, or that it artifactually increased his BMD. Adipose tissue can inflate DXA measurements of BMD, particularly at the spine, hip, and femur, where overestimates can approach 30% (36). By contrast, radial DXA cannot be easily confounded by soft tissue, suggesting that the radius may be a more accurate BMD measurement site for heavier individuals (37). Therefore, the father's radial BMD T-score of −2.6 (age-adjusted Z-score of −2.0) may reflect the pathogenicity of the A745V allele. Finally, and most interestingly, we speculate that the subject's running interacted with his genetics to precipitate his osteoporosis.

The proposition that the A745V polymorphism altered the anabolic response of the subject's bones to mechanical stress not only provides a potential explanation for why his phenotype is more severe than that of his father, but can also explain the peculiar chronology of his fracture history (Figure 2A). If the subject's bones were not able to adapt appropriately to the mechanical stress imposed by distance running, one would expect that he would only begin to experience fractures after a sustained period of habitual distance running, as was indeed the case in our patient. In addition, one would predict that continued distance running would continue to weaken his bones and increase his susceptibility to fracture, as it did. Interestingly, the subject's DXA scan revealed that his lumbar spine, total hip, and femoral neck BMD Z-scores, all of which represent load-bearing sites, were notably lower than his total body (minus head) BMD Z-score (Figure 2B). This observation mirrors the observation that *LRP5* gain-of-function kindred exhibit the greatest increases in BMD at load-bearing sites (30). Notably, the subject's phenotype was more severe at the lumbar spine than at the hip and femur. Counterintuitively, this is also what the mechanotransduction model predicts. Although the spine, hip, and femur are all load-bearing sites, *LRP5* polymorphisms have been reported to alter mechanotransduction in trabecular bone more so than in cortical bone, and the spine has the highest proportion of trabecular bone of these sites (26). The mechanical stress response model is further consistent with data from *LRP5* mouse models, which collectively show that *LRP5* gain-of-function increases bone formation specifically in response to mechanical stress and that *LRP5* loss-of-function reduces the response of bone to mechanical stress in a dose-dependent manner (17, 24, 25, 29). Furthermore, results of the Framingham Cohort and Odense Androgen Studies suggest that *LRP5* polymorphisms can affect the interaction between physical activity and BMD in men, such that men carrying particular polymorphisms do not appear experience the increases in BMD usually associated with weight-bearing activities. Notably, the physical activity data from these studies were limited to self-report questionnaires (31, 32) (Figure 4).

Our report has certain limitations, chief among these being that the subject had no DXA scans available for comparison before his first tibial stress fracture or during his running career. Therefore, we cannot rule out the possibility that the *LRP5* mutation substantially impacted his BMD before the start of his running career, or confirm that his BMD decreased with continued running (as suggested by his decreasing mileage threshold to fracture). We also could not assess the degree to which other factors, such as the subject's ulcerative colitis or nutritional status, may have independently, or by interacting with the A745V variant, contributed to the subject's low BMD. In fact, at the time of diagnosis, when the A745V variant was undocumented and its significance unrecognized, Relative Energy Deficiency in Sport (RED-S) was proposed as a diagnosis of exclusion (33, 34). While the subject's normal BMI (19–22 kg/m^2^) and endocrine assessments made this a less likely diagnosis, it remains possible that insufficient nutritional intake during the high-mileage period of his running career contributed to some extent to his low BMD. Nevertheless, the fact that this young man has osteoporosis, harbors a rare mutation in a gene that is known to modify the response of bone to mechanical stress in animal models (perhaps in a sex-specific manner), and underwent a discrete period of intense mechanical loading during which he became increasingly prone to fracture, suggests that the subject may represent the first documented case of a genetic mutation that contributes to osteoporosis, in part, by altering the anabolic response of bone to mechanical stress. Future work in needed to enhance our understanding of the genetic contributions of *LRP5* to mechanotransduction in bone.

## Ethics Statement

Written informed consent was obtained from the participant for the publication of this case report and any potentially-identifying information/images.

## Author Contributions

All authors listed have made a substantial, direct and intellectual contribution to the work, and approved it for publication.

### Conflict of Interest Statement

The authors declare that the research was conducted in the absence of any commercial or financial relationships that could be construed as a potential conflict of interest.
